# CMR for myocardial characterization in ischemic heart disease: state-of-the-art and future developments

**DOI:** 10.1186/s41747-021-00208-2

**Published:** 2021-03-25

**Authors:** Tilman Emrich, Moritz Halfmann, U. Joseph Schoepf, Karl-Friedrich Kreitner

**Affiliations:** 1 Department of Diagnostic and Interventional Radiology, University Medical Center, Mainz; Langenbeckstraße 1, 55131 Mainz, Germany; 2 German Center for Cardiovascular Research (DZHK), Partner Site Rhine Main, Mainz, Langenbeckstraße 1, 55131 Mainz, Germany; 3 Department of Radiology and Radiological Science, Medical University of South Carolina, 25 Courtenay Drive, Charleston, SC 29425 USA

**Keywords:** Coronary artery disease, Heart, Magnetic resonance imaging, Myocardial infarction, Myocardial ischemia

## Abstract

Ischemic heart disease and its sequelae are one of the major contributors to morbidity and mortality worldwide. Over the last decades, technological developments have strengthened the role of noninvasive imaging for detection, risk stratification, and management of patients with ischemic heart disease. Cardiac magnetic resonance (CMR) imaging incorporates both functional and morphological characterization of the heart to determine presence, acuteness, and severity of ischemic heart disease by evaluating myocardial wall motion and function, the presence and extent of myocardial edema, ischemia, and scarring. Currently established clinical protocols have already demonstrated their diagnostic and prognostic value. Nevertheless, there are emerging imaging technologies that provide additional information based on advanced quantification of imaging biomarkers and improved diagnostic accuracy, therefore potentially allowing reduction or avoidance of contrast and/or stressor agents. The aim of this review is to summarize the current state of the art of CMR imaging for ischemic heart disease and to provide insights into promising future developments.

## Key points

- Ischemic heart disease is a major contributor to mortality and morbidity worldwide.
- Cardiac magnetic resonance (CMR) allows evaluation of regional and global myocardial function.
- CMR provides superior myocardial characterization, including visualization of edema, infarct, microvascular obstruction, and hemorrhage
- Current CMR imaging strategies for perfusion and viability assessment have widely accepted diagnostic and prognostic value.
- Emerging imaging techniques aim to improve diagnostic and prognostic power of CMR.

## Background

Cardiovascular disease remains one of the most common causes of death in industrialized nations [1] with ischemic heart disease (IHD) and its sequelae as the major contributors. On a pathophysiological level, the imbalance between myocardial oxygen supply and consumption causes myocardial ischemia and initiates the ischemic cascade. Initially, starting from the endocardium towards the epicardial layer, the lack of sufficient oxygenation results in ultrastructural changes such as diminished cellular glycogen, relaxed myofibrils, and sarcolemma disruption, which can be seen as early as 10 to 15 min after the onset of ischemia [2]. Such changes may progress into cytogenic and vasogenic edema, which are, in principle, unspecific responses to an acute injury [3]. Finally, unresolved ischemia leads to capillary leakage, membrane flaps, and disruption, resulting in cell necrosis and initiation of repair strategies, such as inflammation and deposition of granulation tissue. The process eventually culminates in scar formation. Clinically, in the acute stage, these processes can lead to potentially lethal complications such as severe arrhythmias and cardiogenic shock due to electrical instability and impaired myocardial contractility. In the subacute stage, myocardial rupture may occur. At the chronic stage, IHD is one of the major reasons for the development of congestive heart failure.

Over the last decade, technological advances have strengthened the role of noninvasive cardiovascular imaging techniques for the detection, quantification, and risk stratification in acute and chronic IHD. Alongside echocardiography, nuclear medicine, and computed tomography, cardiac magnetic resonance (CMR) has evolved as an essential tool for the characterization of IHD [4, 5]. In addition to objective measurements of left ventricular (LV) structure and function, CMR provides the ability to visualize myocardial ischemia, the following acute injury, and potential chronic replacement fibrosis.

In general, evaluation of IHD with CMR relies on four major pillars that have different roles depending on the progression of the disease:

A. *Functional analysis* to evaluate wall motion disturbances and ventricular function;
B. *Edema imaging* to differentiate between acute and chronic myocardial injuries;
C. *Stress imaging* to detect inducible ischemia;
D. *Infarct imaging* to visualize and quantify permanent myocardial injury

CMR allows for the detection of subclinical IHD (A + C) or clinically evident IHD (A, B, D), and risk stratification in patients suffering from acute or chronic IHD [6]. The aim of this review article is to summarize the current state-of-the-art CMR knowledge in IHD and to provide a glimpse into current obstacles and promising future CMR applications.

## Functional analysis to evaluate wall motion disturbances and ventricular function

Analysis of myocardial wall motion and ventricular volumes with cine sequences is a basic CMR concept and a widely accepted reference standard for the evaluation of left and right myocardial wall motion and ventricular function [7]. IHD leads to impairment of wall motion in various degrees. Mild degrees of ischemia typically result in hypokinetic areas in the myocardium; with increasing ischemic damage, hypokinesia may progress into akinesia. In late stage IHD, myocardial damage can lead to paradoxical motion, *i.e.,* dyskinesia. A short period of myocardial ischemia (< 15 min) is able to induce transient ventricular dysfunction (the so-called *stunned myocardium*), which can be present for hours or even up to days, even in the event of timely restoration of coronary blood flow. In contrast, the term *hibernating myocardium* describes the effect of a longer lasting or repetitive exposure of the myocardium to an ischemic state. This results in partial or complete myocardial contractile dysfunction, which can be potentially reversed by revascularization. Finally, if myocardial ischemia is too severe or prolonged, myocardial necrosis will lead to irreversible loss of myocardial contractile function [8].

The clinical assessment of wall motion is mostly performed on a visual basis that does not allow quantification of wall motion impairment. Changes in wall thickness may serve as a basic tool to measure wall motion abnormalities. However, this method is time-consuming and has not been accepted and implemented widely in the clinical routine [9]. Cine imaging-based LV function reflects a cornerstone in the evaluation of IHD. Several studies have shown the value of ejection fraction to predict risk in acute and chronic IHD as well as guiding of therapy in regard to the need of implantable cardioverter defibrillator and cardiac resynchronization therapy systems [10, 11].

Standard cine imaging requires the collection of image data over several heart beats and results in a “segmented” acquisition of a single cardiac plane, typically using a balanced steady-state free precession (bSSFP) or fast gradient echo technique [12]. Therefore, segmented cine imaging is susceptible to (tachy-)arrhythmia and breathing-related artifacts. To mitigate these problems, various acceleration techniques have been proposed to improve the time efficiency of image acquisition such as compressed sensing and “real-time” imaging, which allow the acquisition of cine images of the whole heart in a single breath hold. Compressed sensing strategies are based on the incoherent under sampling of *k*-space, transformation of sparsity, and iterative nonlinear reconstruction. Therefore, compressed sensing allows for up to 40-fold acceleration while still providing accurate assessment of LV function [13].

To obtain quantifiable information about wall motion, the concept of *strain imaging* has been established. Early applications needed the acquisition of special “tagged” sequences that allow the visualization and quantification of myocardial deformation on CMR images. Alongside further developments, such as “displacement encoding with stimulated echoes and strain-encoded” CMR, the application of feature tracking algorithms on regular cine images helped strain imaging to gain attention in clinical routine [14]. Therefore, feature tracking-based strain calculations are theoretically easy to implement into clinical workflows, as they utilize routinely acquired cine sequences and myocardial segmentation for functional analysis. Other techniques, such as the “displacement encoding with stimulated echoes and strain-encoded” calculations acquisition and post-processing need dedicated research sequences and software tools for strain evaluation.

**Fig. 1:**
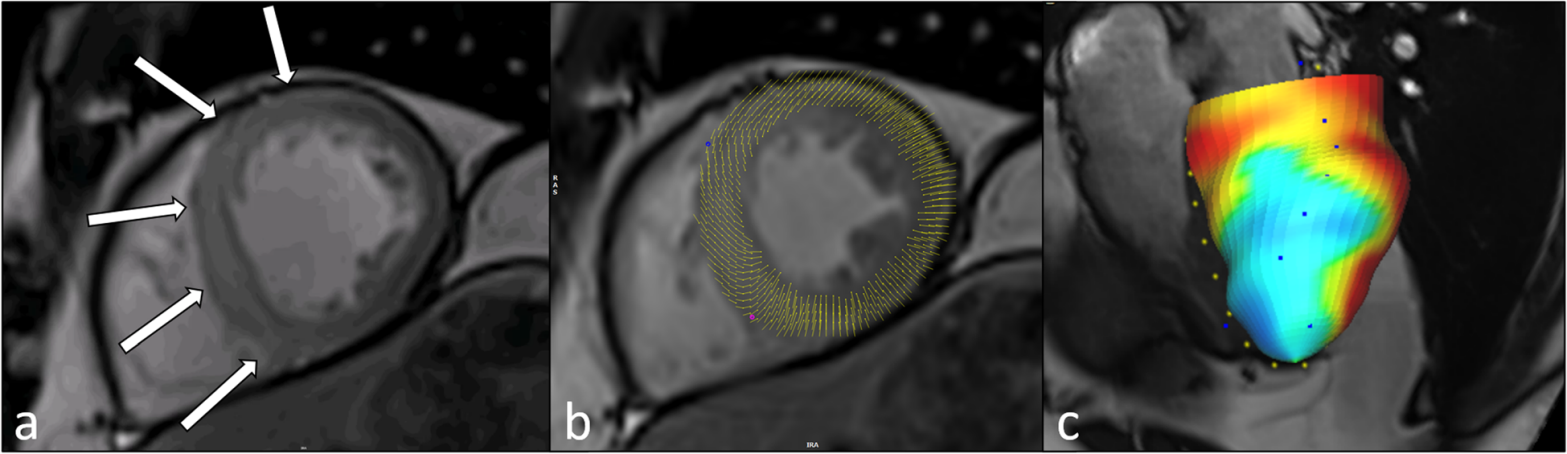
Thirty-two-year-old man with acute anteroseptal myocardial infarction. Diastolic phase, post-contrast cine bSSFP image indicates areas of myocardial edema and wall motion abnormalities (**a**, white arrows). Systolic phase image (**b**) is shown with myocardial displacement overlay. 3D visualization with Feature Tracking radial strain overlay indicates reduced contractility (**c**, blue area) in the affected territory. *bSSFP* Balanced steady-state free precession

In summary, strain imaging is able to determine myocardial deformation over the cardiac cycle. Cardiac deformation during contraction can be subdivided into three principal motion patterns: longitudinal and circumferential shortening and radial increase of wall thickness. Each motion pattern is related to a different myocardial layer, which allows the derivation of the location and severity of the impairment [15]. Strain imaging has shown its incremental value to improve diagnosis and quantification of wall motion disturbances in acute and chronic ischemic cardiomyopathy in preclinical animal models [16] and human studies [17–19]. Recent publications demonstrated the incremental prognostic value of strain imaging in risk stratification after myocardial infarction [20–23]. Nevertheless, there is neither established consensus nor standardization for strain imaging as different post-processing tools use different algorithms to calculate strain parameters. In addition, strain values are not interchangeable among different software solutions and reproducibility of strain measurements is dependent on the software used, as well as the type of myocardial strain, with global radial strain as the least reproducible global strain parameter [24]. An example of feature tracking-based analysis of wall motion abnormalities is shown in Fig. 1. It is highly anticipated that automated functional and wall motion analysis becomes simpler, faster, more widely available, and potentially enter clinical reality, as novel artificial intelligence convolutional neural network and machine learning-based tools have been developed [25, 26] and showed promising applications, *e.g.*, for segmenting CMR imaging data [27].

## Edema imaging to differentiate between acute and chronic myocardial injuries

Discrimination between acute and chronic infarcts relies on the capability of CMR to detect myocardial edema. Acute ischemic damage leads to cell death and inflammatory response, which leads to accumulation of water in the affected territory. Such territory is usually referred to as the area-at-risk (AAR), *i.e.*, the area affected by the occlusion of a coronary artery [28]. Within the AAR lies the infarcted zone containing necrotic, irreversible damaged tissue which may expand into the surrounding edematous AAR, if coronary reperfusion is not established in a timely manner. The ratio between infarcted zone and AAR allows for the description of the myocardial salvage index [29]. This index has been shown to be an independent predictor for the occurrence of major adverse cardiac events (MACE) [30].

**Fig. 2:**
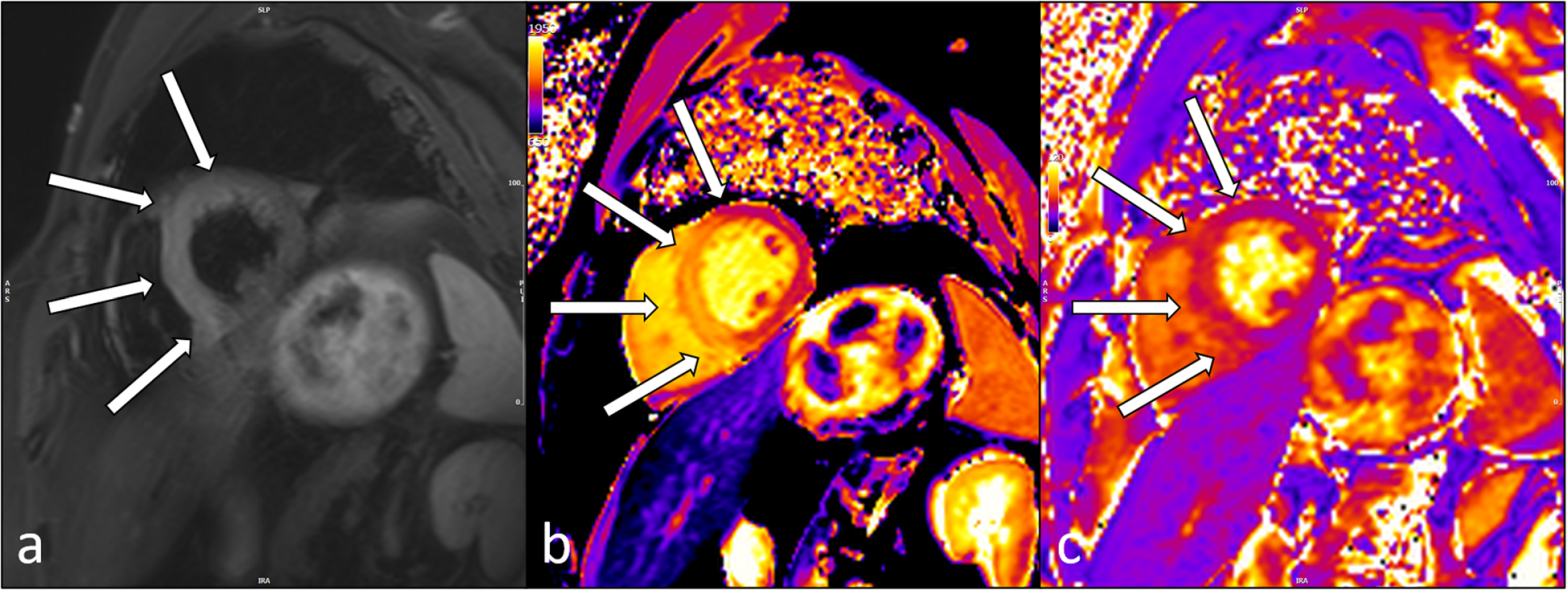
Thirty-two-year-old man with acute anteroseptal myocardial infarction (same patient as in Fig. 1). T2-STIR image shows excessive edema (**a**, white arrows), corresponding to increased native T1 (**b**, white arrows) and T2 (**c**, white arrows) relaxation times in the affected myocardium. T2-STIR, T2-weighted short-tau inversion recovery

Conventional T2-weighted images are preferred for the detection of myocardial edema compared with conventional T1-weighted images [31]. In clinical routine, the T2-weighted short-tau inversion recovery (STIR) technique is the most commonly used because of its availability by all major vendors [32, 33]. Typically, these sequences show areas of increased signal intensity in the edematous myocardium in comparison to remote myocardium (Fig. 2). Optimal contrast is achieved by using triple inversion to suppress signals from both fat tissue and flowing blood. However, T2-weighted sequences are prone to bright-blood artifacts (due to incomplete blood suppression) close to the endocardial border due to stagnant and/or slow blood flow, and breathing and motion artifacts due to long acquisition times and surface coil intensity variation. Further disadvantages of this sequence types include the lack of quantitative analysis and the need for “normal” tissue reference [34]. Overall, these obstacles can lead to insufficient diagnostic value in over 20% of cases [35].

Alternatively, the contrast-enhanced cine bSSFP (CE-bSSFP) technique has been investigated to evaluate myocardial edema in myocardial infarction (see Fig. 1). CE-bSSFP relies on the change in the T2/T1 signal in injured myocardium in comparison with remote myocardium [36]. This approach has been shown to be less sensitive to image artifacts, superior to T2-weighted STIR for detection of the culprit lesion [37], and demonstrated good correlation with the histopathologic extent of AAR [38]. However, CE-bSSFP relies on the accumulation of gadolinium-based contrast agent, which can be insufficient in total coronary occlusion without reperfusion.

Over the last years, T1 and T2 mapping techniques have emerged as promising myocardial characterization tools [39, 40]. There are numerous approaches for the quantification of T1 relaxation times, most of them using a single-shot bSSFP acquisition with inversion preparation (*e.g.*, modified Look-Locker inversion recovery, MOLLI), shortened MOLLI (ShMOLLI) [41, 42], saturation preparation (*e.g.*, saturation recovery single-shot acquisition) [43], or the combination of both (saturation pulse prepared heart-rate independent inversion recovery) [44]. In clinical routine, MOLLI has become widely used due to its excellent precision. However, MOLLI-based T1 estimation is sensitive to magnetization transfer [45], T2 effects, inversion pulse efficiency, heart rate (variability), and off-resonance effects, which may reduce its accuracy.

T2 mapping is performed by pixel-wise fitting of a T2 decay curve from a series of T2-weighted images. These images can be acquired by different sequence types, *e.g.*, by turbo spin echo sequences with varying echo times [46], a bSSFP or spoiled gradient echo with initial T2 preparation module [47, 48], or a sequence scheme that combines turbo spin-echo excitation and gradient-echo readout (gradient and spin echo, GRaSE) [49]. All of these techniques provide images with different echo times that can be used to estimate T2 values by two- or three-parameter model fitting.

Myocardial edema will prolong native T1 as well as T2 relaxation times (Fig. 2). In brief, T1 mapping is sensitive to detect myocardial edema, but T2 mapping is more specific as T1 relaxation times are also prolonged by the presence of excessive water as well as other tissues such as fibrotic tissue and blood. Animal and clinical studies have shown good correlation of T1 and T2 mapping to the AAR measured by microspheres [50–52]. In direct comparison, native T1 mapping using a ShMOLLI sequence was superior to T2-weighted imaging in non-ST-elevation myocardial infarction and equal to T2-weighted imaging in ST-elevation myocardial infarction (STEMI) [53]. T2 mapping can also be used to track the resolution of myocardial edema [54] in a quantitative manner. Moreover, additional tissue changes in infarction such as hemorrhage and chronic scar can complicate the analysis of T2-weighted imaging and parametric mapping to detect myocardial edema, *e.g.*, by blood degradation products such as deoxyhemoglobin and methemoglobin induce a decrease in T2 in the infarct core [55]. Nevertheless, there is no current standard and reference sequence for T1 or T2 mapping that is widely accepted and distributed. However, standardization initiatives such as the “T1MES” phantom project are promising approaches to increase quality assurance of mapping sequences [56].

## Stress imaging to detect inducible ischemia

Stress myocardial perfusion CMR has proven its reliability for the detection of stress-inducible ischemia in suspected IHD. CMR-based assessment of inducible ischemia has been shown as a cost-effective technique, applying a high spatial resolution multiparametric approach that has the ability to discriminate ischemia and normal myocardium [57]. Currently, there are two basic concepts which allow imaging of inducible ischemia with CMR, both rely on the administration of pharmacological stress agents.

**Fig. 3:**
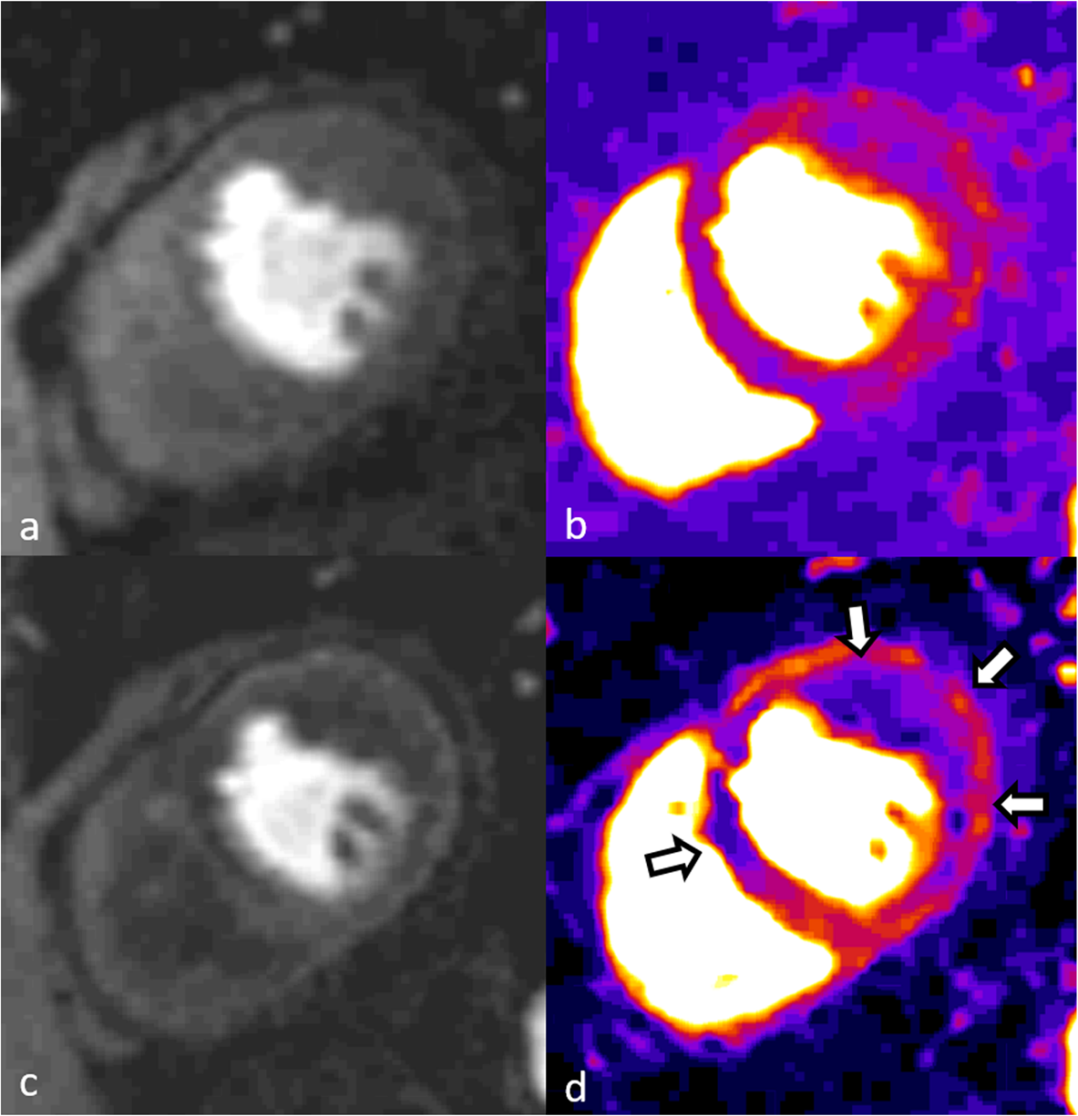
First pass perfusion at rest (**a**) and stress (**c**) in a 58-year-old man with suspected coronary artery disease. Semiquantitative parametric slope maps (**b**) show no perfusion defects at rest, while extensive perfusion deficit (white arrows) in the anterior septum (left anterior descending area) and lateral wall (circumflex area) is demonstrated at stress (**d**), indicating transient ischemia likely due to left main disease

First, vasodilators such as adenosine or regadenoson are used as they increase blood flow in more “normal” coronary arteries but not in severely stenotic arteries as the depending microcirculation that a stenotic artery supplies is already maximally dilated. During maximal stress, a contrast-enhanced perfusion acquisition is performed. A typical perfusion defect is visualized as an endocardial to transmural dark zone while the rest of the myocardium is enhanced by the contrast agent (Fig. 3). In contrast, a dark rim artifact is detectable as a transient endocardial low signal in the early phases of the first pass perfusion, which typically fades out at the time point of the myocardial enhancement. Several mechanisms have been proposed to explain the presence of such artifact, including Gibbs ringing in the phase encoding direction at the blood–myocardial border, magnetic susceptibility associated with the gadolinium bolus or partial volume effects [58]. The dynamic nature of perfusion studies allows for the detection of time-resolved changes in myocardial perfusion. Semiquantitative evaluation can be used to calculate an indexed ratio of the upslopes of the perfusion-time intensity curves in response to vasodilator stress compared with the rest scan (myocardial perfusion reserve index) [59]. Stress myocardial perfusion has been shown to have better sensitivity and negative predictive value compared with single photon emission computed tomography (SPECT), excellent accuracy to detect single- and multi-vessel coronary artery disease [60], and to deliver comparable results to positron emission tomography-based estimation of myocardial perfusion [61]. A recent investigation by Nagel et al. [62] has also demonstrated its noninferiority to predict MACE in comparison with invasive fractional flow reserve.

Second, positive inotropes and chronotropes such as dobutamine can be administered to increase myocardial oxygen demand comparable to physical exercising [63]. Dobutamine can be given at a low dose to recruit the contractile reserve of hibernating myocardium (*i.e.*, functionally impaired but viable myocardium) or a high dose to induce myocardial ischemia in territories with significant coronary stenosis that can be depicted by the onset of wall-motion abnormalities. High-dose dobutamine CMR is superior to dobutamine stress echo in regard to diagnostic accuracy [64]. It has been demonstrated that the presence of inducible ischemia or an ejection fraction of < 40% independently predicts future myocardial infarction or cardiac death [65]. Korosoglou et al. [66] have shown that wall motion abnormalities during stress have strong independent prognostic value for MACE and late revascularization, while patients with normal wall motion at stress have very low risk for future MACE.

Both stress techniques, however, have limitations. The administration of vasodilators or inotropes carries the risk of potential life-threatening side effects. Imaging at stress has to be performed at the optimal time point when the stress effect is maximized. Furthermore, conventional adenosine and dobutamine stress images are currently analyzed by semiquantitative methods, limiting the evaluation to visual detection (“eyeballing”) which also requires the identification of remote tissue to compare pathological changes with [9]. Consequently, diffuse pathologies such as microvascular disease can be missed by conventional approaches.

Irrespective of the accumulating evidence for the value of stress CMR, new developments promise to overcome its current limitations. One major goal is to achieve capability of acquiring fully quantitative perfusion data sets [67], preferably in a pixel-wise fashion, where a parametric myocardial blood flow map is obtained. Recently, a quantitative perfusion approach has gained scientific interest [68]; this technique allows to quantify myocardial blood flow and myocardial perfusion reserve. However, this approach requires a dedicated scan protocol including specific injection rate, contrast medium, and the acquisition of arterial input function. First applications of quantitative myocardial perfusion have shown promising results to independently predict outcome [69]. Additionally, such quantitative analysis has the potential to overcome disadvantages related to qualitative evaluation [70]. Other groups have investigated the accuracy of other automatic pre-processing of perfusion studies [71] and developed a phantom for pixel-wise validation of quantitative perfusion studies [72]. Despite promising first applications, these techniques have not been evaluated yet in multicenter settings, necessary for their implementation in clinical routine.

For dobutamine stress CMR, strain imaging has been implemented to overcome the limitations of visual analysis when evaluating wall motion abnormalities. Korosoglou et al. [73] have shown that strain-encoded CMR improves the diagnostic accuracy for the detection of inducible ischemia during peak dobutamine stress (98% *versus* 83%). Schneeweis et al. [74] demonstrated the use of feature tracking-based strain imaging during dobutamine stress. Furthermore, the use of strain imaging has been shown to reduce inter-observer variability for the detection of wall motion abnormalities [75]. Finally, blunted global longitudinal strain at stress has been shown to be an independent predictor of MACE in patients with known or suspected coronary artery disease with incremental value to standard clinical and imaging risk factors [23].

As an alternative, stress imaging without the need for pharmacological agent and/or contrast media administration has moved into the current scientific focus.

Exercise CMR using MRI compatible treadmills or mountable bikes is a promising method, when contraindications and intolerable side effects prevent the use of a pharmacological stress agent [76]. Several studies have investigated exercise CMR in coronary artery disease with analysis of regional wall motion abnormalities and/or perfusion imaging, demonstrating feasibility, good diagnostic performance, and prognostic implications [77–80]. However, clinical-use exercise CMR is currently restricted, due to limitations such as difficulties of image acquisition and quality, technical challenges, and expenses for commercially available CMR compatible exercise devices [76].

Stress T1 mapping is a novel way to perform stress imaging without the need for administering contrast media, as it relies on the capability of native T1 mapping to detect changes in myocardial blood volume. Physiological response to vasodilators leads to an increase in myocardial blood volume. Therefore, T1 maps acquired before and during vasodilator stress show a difference in myocardial T1 due to the increased blood volume in the myocardium [81]. Notably, this technique relies on accurate measurements of T1 relaxation times, especially at high heart rates under stress.

Another experimental approach to avoid contrast administration for stress imaging is *arterial spin labeling*. It relies on the tracing of “labeled” water protons by changing their longitudinal magnetization using selective radiofrequency pulses and compare images with and without the inflow of labeled blood. This technique has been evaluated in various animal models of myocardial ischemia and infarction [82, 83], and humans [84]. Currently, there are several drawbacks, such as low signal-to-noise ratio, limited reproducibility, and issues with motion correction and image registration [85], which limit its clinical implementation.

Finally, the combination of blood-oxygen-level-dependent imaging with breathing maneuvers has been assessed for the indirect evaluation of myocardial perfusion [86]. Deoxygenated hemoglobin serves as intrinsic contrast medium, that can be measured by T2 and T2* mapping. Hyperventilation and apnea, that modulate blood carbon dioxide levels, can be used as a potent vasodilator. This approach has been evaluated in healthy volunteers [87], coronary artery disease [88], and hypertension patients [89]; however, no large cohort validation has been performed to date.

## Infarct imaging to visualize and quantify permanent myocardial injury

Imaging of necrotic tissue (acute infarct) and scar (chronic infarct) is a hallmark of clinical CMR. However, as infarcts may have a diverse histology depending on severity, presence of hemorrhage and/or microvascular obstruction, and changes over the time from coagulation necrosis to mature scar, imaging characteristics may vary. Infarct imaging is typically done using late gadolinium enhancement (LGE) CMR. The basic principle behind LGE is demonstrating the distribution of gadolinium-based contrast agent that normally accumulates in the extracellular space, but, in case of infarction, it can enter the intracellular space through ruptured cell membranes. The extent of gadolinium accumulation is related to the blood volume, tissue perfusion, size of the extracellular space, and amount of necrotic tissue. The LGE phenomenon is caused by delayed gadolinium washout from the diseased *versus* healthy myocardium. As gadolinium-based contrast agent causes T1 shortening, diseased myocardium appears bright on T1-weighted images. Typically, conventional inversion recovery gradient-echo sequences are used to acquire LGE images. However, a phase-sensitive inversion recovery (PSIR) technique is also available that reduces the need of precise inversion times and provides more consistent signal and contrast. In addition, “synthetic” LGE images can be retrospectively generated from post-contrast T1 maps and allow LGE image evaluation at any theoretical inversion time [90].

Accuracy of CMR to detect and quantify infarct size has been validated against histopathology in animal studies [91]. Its excellent spatial resolution allows for the detection of small areas of infarct [92, 93], that outperforms SPECT, especially in cases with non-anteriorly located infarcts [94]. LGE is the key discriminator between ischemic and nonischemic cardiomyopathies [95] and a reliable tool for assessment of myocardial viability and likelihood of recovery after revascularization [96]. A transmurality less than < 50% has been shown to predict a very high likelihood for functional recovery, while functional improvement in segments with scar transmurality of > 50% was only 8% [97].

To date, there is no universally accepted method for LGE quantification. Several techniques have been suggested including manual contouring and semi-automated approaches using signal intensity thresholds of 2 to 6 times the standard deviation of the signal from the normal myocardium, the full-width-at-half-maximum approach, and nonbinary techniques [98, 99]. Most of these methods are time-consuming, require the definition of regions of interest in remote and/or enhanced myocardium, and different methods lead to somewhat varying results [100]. Therefore, precise and fully automated objective LGE quantification remains a research tool without current clinical implementation.

LGE imaging also provides insights into irreversible damage of the microvascular circulation by visualizing microvascular obstruction (MVO), also known as no-reflow phenomenon, and intramyocardial hemorrhage (IMH). Percutaneous revascularization typically aims to restore epicardial blood flow, but may not improve microvascular perfusion. Insufficient microvascular perfusion affects approximately 50% of STEMI patients and manifests as MVO and subsequent IMH [101]. It has been shown that the capillaries are plugged by erythrocytes, platelets, and microthrombi and lined by swollen endothelial cells. Hypoxia-induced destruction of the endothelial barrier leads to extravasation of blood cells upon reperfusion, resulting in IMH [102]. MVO and IMH can be identified as low or absent signal areas in LGE images, typically located within the central portions of the infarcted tissue, which are usually large infarcts, with otherwise bright signal on LGE. MVO can be quantified by manual planimetry. Alternatively, MVO can be diagnosed on T2-based imaging as T2* mapping or T_2_-STIR.

The assessment of the extent of nonviable tissue in IHD has significant prognostic value. A meta-analysis incorporating 2,632 patients from 10 randomized trials found that a 5% increase in infarct size correlates to a 20% increase in rates for heart failure-related hospitalization and all-cause mortality [103]. Furthermore, LGE extent was a strong predictor of MACE, independent from LV function [96]. MVO was a stronger independent predictor of LV dysfunction and post-infarct complications compared to LGE extent [104, 105], and infarct size and MVO outperformed clinical risk scores and LV ejection fraction [6, 105]. MVO most likely reflects irreversible tissue destruction and clinically relevant reperfusion injury, which has been shown to have a strong association with negative LV remodeling [106]. Thus, besides quantifying the size of the infarct, the evaluation of vascular integrity may have a pivotal role in the outcome prediction of acute myocardial infarction.

**Fig. 4:**
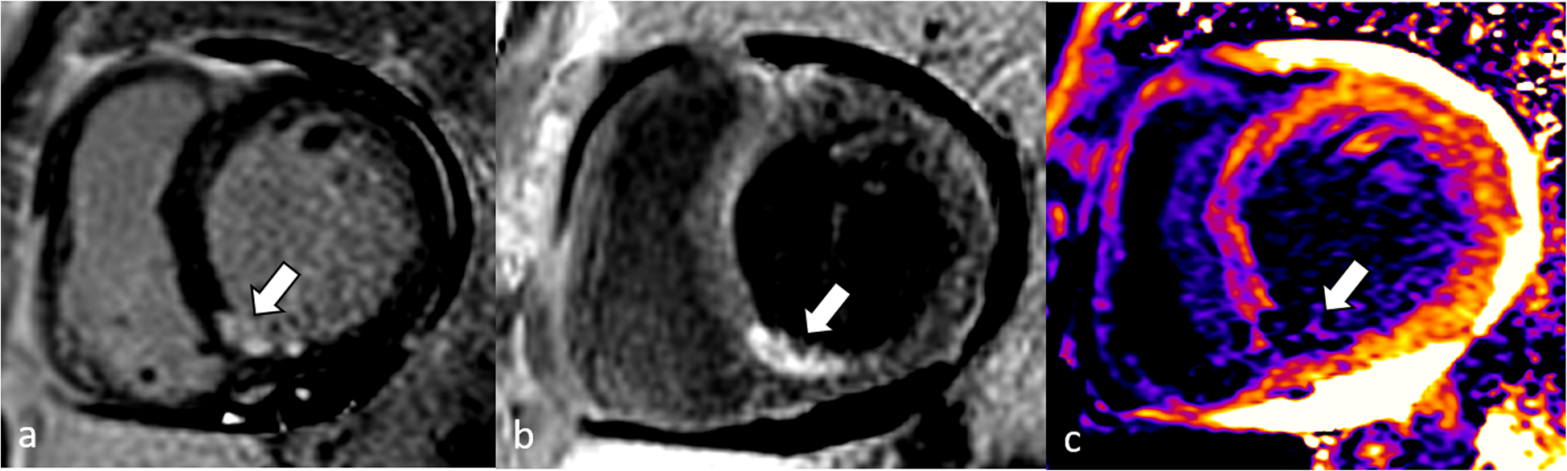
Conventional LGE (**a**), dark blood LGE (**b**), according Muscogiuri et al [108], and T1 map (**c**) are shown in a 62-year-old male with known coronary artery disease. Conventional LGE shows small area of hyperenhancement in the inferoseptal and inferior segments (white arrow), corresponding to myocardial infarct in the right coronary artery territory. The same area is depicted by dark-blood LGE and the T1 map (white arrows). Note that the discrimination of blood and infarct signals is improved on dark-blood LGE compared with the conventional technique. *LGE* Late gadolinium enhancement

**Fig. 5:**
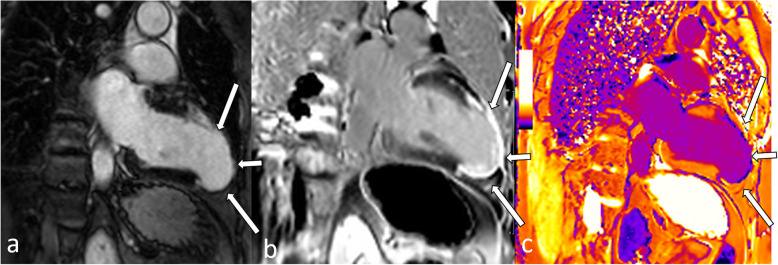
Eighty-year-old woman with chronic antero-apical myocardial infarction. The affected left ventricular territory is shown by blue arrows in the corresponding conventional LGE image (**a**), dark-blood LGE image (**b**), according to Holtackers et al. [110], and post-contrast T1 map (**c**). *LGE* Late gadolinium enhancement

Despite the promising diagnostic and prognostic implications, there are multiple limitations to the current LGE techniques. Due to the similarity between enhancing infarct and blood pool signals, small subendocardial enhancements can sometimes be missed. To overcome this problem, so-called black-blood or dark-blood LGE sequences have been proposed that allow for the visualization of sufficient tissue contrast while simultaneously suppressing blood pool signal, such as the “flow-independent dark-blood delayed enhancement” [107], and the “T(rho) and magnetization transfer and inversion recovery” [108] techniques. Kellman et al. [109] proposed a similar method that combines inversion recovery and T2 preparation with single-shot SSFP imaging. While most of these techniques have shown superiority to conventional LGE for the detection of subtle subendocardial myocardial infarcts [108–110], all of them require additional magnetization preparation modules / pulses that are not generally available. A novel approach described by Holtackers et al. [110], however, is built on the clinically available PSIR LGE sequence. By setting the inversion time for blood-pool nulling instead of myocardium nulling in a PSIR LGE sequence, suppression of the blood signal is achieved without compromising the hyperenhancement of scar tissue. The nulled blood pool appears black in the magnitude image, but appears mid-gray in the PSIR image as the healthy myocardium has an even lower (*i.e.*, more negative) magnetization level, appearing black in the PSIR image. As the hyperenhanced scar regions have shorter T1 relaxation times compared with the blood pool, they still appear bright in the PSIR image. Validation of this approach has shown promising results across scanners from different vendors on both 1.5-T and 3-T magnets [111]. Image examples comparing conventional LGE with black-blood or dark-blood-LGE and T1 mapping are shown in Figs. 4 and 5.

Another general limitation of LGE imaging is that diffuse myocardial involvement is difficult to quantify (*e.g.*, infiltrative diseases or microscopic fibrosis), as myocardial enhancement has to be evaluated relative to reference normal tissue. Mapping techniques, however, measure intrinsic tissue properties, therefore do not rely on reference tissue, thus are capable of assessing diffuse processes such as inflammation in myocarditis or fibrosis in dilated cardiomyopathy. T1 mapping is sensitive to changes in the composition of cellular and interstitial components such as water, protein, fat, and fibrotic tissue. This combination limits the use of native T1 mapping to detect and quantify the extent of myocardial infarct, as edema and infarct may both lead to similar changes in T1 [112]. As an alternative, the combination of native and post-contrast blood and myocardial T1 measurements adjusted with hematocrit allows the calculation of extracellular volume (ECV). The ultimate reason to implement mapping, especially ECV, for the assessment of IHD lies in (a) the possibility to better quantify the extent of infarct based on absolute quantification of ECV and (b) assess diffuse damage of the remote myocardium, which could be an important predictor for recovery and positive ventricular remodeling [113]. For this purpose, future advances in mapping sequences are expected to allow for whole LV myocardium coverage. In addition, T1 and T2 mapping are also useful to determine IMH and AAR [52, 114]. As demonstrated by Bulluck et al. [52], a hypointense core in the AAR visualized by T1 and T2 maps detected the presence of IMH with good sensitivity and specificity.

A currently experimental, but potentially promising approach for CMR scar imaging is the use of fibrosis-specific contrast agents [115]. These types of contrast agents enrich the pool of conventional contrast agents with molecular imaging aspects and promise to provide insights into the early-disease stages and steps of scar evolution. For example, EP-3533 is a peptide-based gadolinium probe specific for type I collagen, therefore allows selective imaging of collagen, a main tissue contributor in scar formation [116]. Helm et al. have demonstrated the use of such contrast agent in a mouse model with myocardial infarction in comparison to histology [117]. Annexin-V is a marker expressed in apoptotic cells and has been used as target for noninvasive imaging of apoptosis after myocardial infarction. AnxCLIO-Cy5.5 is an Annexin-V-labeled nanoparticle that has been investigated in a murine model of acute ischemia [118] targeting necrosis and apoptosis. Other groups have demonstrated the use of molecular imaging in combination with CMR to target healing after myocardial infarction [119] and maturation of scar [120]. Molecular imaging targeting other aspects of fibrosis such as matricellular proteins or matrix-degrading enzymes have been developed but mainly linked to other imaging modalities including positron emission tomography or SPECT; however, no CMR-based molecular imaging method for cardiac fibrosis has made the transition from an experimental phase to a clinical tool [121].

## Future outlook and conclusions

Current clinical CMR methods for anatomical and functional imaging of the myocardium in IHD are well established and have proven their diagnostic and prognostic power over the last three decades. Despite these promising applications, most of the traditional methods are unable to provide precise automated quantification of tissue characteristics (myocardial blood flow, severity and extent of edema, viability, IMH, MVO). Therefore, novel quantitative techniques, such as relaxation mapping, strain imaging, and quantitative perfusion are emerging and en route to being integrated into clinical workflow and have the potential to improve diagnostic and prognostic performance of CMR in the future. Potential future applications including machine-learning approaches, use of physiologic stressors, and/or the use of molecular imaging in combination with CMR have to be translated from experimental approaches to clinical applications in the future.

## Data Availability

Not applicable

## Abbreviations

AAR: Area at risk
bSSFP: Balanced steady-state free precession
CMR: Cardiac magnetic resonance
ECV: Extracellular volume
IHD: Ischemic heart disease
IMH: Intramyocardial hemorrhage
LGE: Late gadolinium enhancement
LV: Left ventricle
MACE: Major adverse cardiac events
MOLLI: Modified Look-Locker inversion recovery
MVO: Microvascular obstruction
PSIR: Phase-sensitive inversion recovery
ShMOLLI: Shortened MOLLI
SPECT: Single photon emission computed tomography
STEMI: ST elevation myocardial infarction
STIR: Short-tau inversion recovery
